# Torsional Mode Phacoemulsification: Effective, Safe Cataract Surgery Technique of the Future

**DOI:** 10.4103/0974-9233.61220

**Published:** 2010

**Authors:** Ahmed M. El-Moatassem Kotb, Mohamed M. Gamil

**Affiliations:** 1 Department of Ophthalmology, Ain-Shams University, Cairo, Egypt; 2 Ain Shams University,Cairo, Egypt. Consultant cataract and glaucoma, Mouwasat Hospital, Kingdom of Saudi Arabia

**Keywords:** Cataract Densities, Longitudinal Mode Phacoemulsification, Torsional Mode

## Abstract

**Purpose:**

To compare various outcome measures using torsional mode and longitudinal mode in the phacoemulsification of cataract with different nuclear densities.

**Setting:**

Magrabi Eye Hospitals, Kingdom of Saudi Arabia.

**Design:**

A randomized comparative clinical study.

**Materials and Methods:**

This study includes 200 eyes of 156 patients (100 in the ultrasound longitudinal “US” group and 100 in the torsional group). All eyes received AcrySof^®^ single piece intraocular lens (Alcon Surgical, Fort Worth, TX). The primary outcome measures were ultrasound time (UST), cumulative dissipated energy (CDE), and surgical complications. Postoperative outcome measures were the degree of corneal edema on the first postoperative day and final best corrected visual acuity (BCVA) and CCT (central corneal thickness).

**Results:**

The differences in UST and CDE between subgroups of nucleus hardness were statistically significant (*P* < 0.01). The UST and CDE consistently increased in eyes with higher grades of nucleus density. On day one, the mean BCVA was 0.61 ± 0.13 decimals in the ultrasound (US) group and 0.67 ± 0.11 decimals in the torsional group (significant *P* < 0.05). Corneal edema was significantly less in the torsional group (*P* < 0.05). At 30 days, the mean BCVA was 0.94 ± 0.22 decimals in the US group and 1.0 ± 0.12 decimals in the torsional group but this difference was not statistically different (*P* > 0.05).

**Conclusions:**

The torsional mode provides an effective and safe method for cataract removal with lower energy usage as compared to longitudinal traditional phacoemulsification. However, the final visual outcome was similar for both study groups.

## INTRODUCTION

Advancement in phacoemulsification technology allows use of smaller surgical instruments, flexible intraocular lenses (IOL) and advanced management software for phacoemulsification units further reducing incision size and tissue trauma and promoting faster functional recovery. In conventional US mode, the US power to emulsify the lens is derived from the longitudinal excursion of the phacoemulsification needle.^1^ Phacoemulsification tip moves for ward and backward at a high frequency. However, in the conventional US mode, phacoemulsification can produce repulsion effect because the phaco tip pushes the nucleus away with each stroke as it moves forward. Thus, the US is interrupted and efficiency of phacoemulsification is compromised.^2^

In January 2006, Alcon Surgical incorporated OZil torsional into the Infiniti Vision System. The OZil torsional portion is a hardware and software upgrade of the machine and includes a dedicated hand-piece that produces rotary oscillations of the phacoemulsification tip with a frequency of 32 KHz. It is suggested that the OZil torsional oscillation effect and the incorporated improvements reduce the amount of phacoemulsification energy and increase the efficiency required to remove the cataractous nucleus because it does not produce repulsion and breaks up cataract by shearing and not by conventional jackhammer effect.^2^

Improvements in phacoemulsification surgery afforded by torsional phacoemulsification are found in the OZil hand-piece's unique movement profile and reduced frequency.^3^ The shaft of the torsional hand-piece oscillates, and the oscillatory motion is translated to side-to-side movement at the tip. Torsional phacoemulsification uses angulated tips, so the motion of the tip within the nuclear tissue is up to three times greater than movement of the tip's shaft, according to Dr. Mackool.^3^

We designed this study to compare different clinical outcomes using torsional versus longitudinal phacoemulsification mode in different cataract densities.

## MATERIALS AND METHODS

### Participants

Patients aged 54 years or older, with the diagnosis of age-related cataract, have been included in the study. Patients who had other ocular or systemic disorders affecting vision were excluded. These included patients with diabetic retinopathy, glaucoma, age-related macular degeneration, uveitis, corneal endothelial disease or previous intraocular surgery.

### Methods and surgical technique

First, patients are enrolled and informed consent is obtained from them. Standard preoperative ophthalmological examinations including lens nucleus density grading according to the Lens OpacitiesClassification System II (LOCS II)^4^ are then performed.

### Surgical technique

All patients received periocular anesthesia^5^ and surgeries were performed by the same surgeon (A.K.). A 2.8 mm self sealing limbal incision was made at 12 O'clock and a paracentesis using 15° blades performed at about 60° from the main incision. Sodium hyaluronate 3.0%- chondroitin sulfate 4.0% (Viscoat, Alcon Surgical, Fort Worth, TX) was used to reform and stabilize the surgical planes and protect the corneal endothelium. A 5.5 to 6.0 mm continuous curvilinear capsulorhexis was performed with a bent 27-gauge needle.

A standard horizontal chopping technique^6^ was used with the Infiniti Vision System with the US pulse mode or torsional mode. We used the OZil torsional hand-piece through a 2.8 mm incision (the 0.9 mm tapered Kelman microtip with a 30° bevel [Alcon Laboratories, Inc. Fort Worth, TX]).

**Figure 1:**
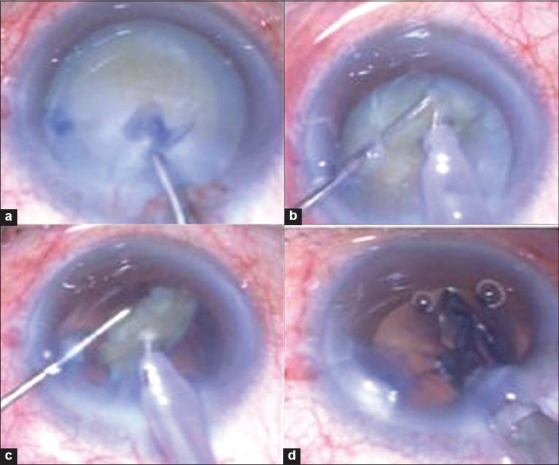
Torsional phacoemulsification in mature cataract (grade 4 nucleus density). a. Finalizing the capsulorhexis, b. Horizontal chopping technique, c. Last piece phacoemulsification, d. IOL implantation

The surgical parameters used in either group are summarized in Table 1. An intraocular lens AcrySof^®^ Single Piece Intraocular Lens (Alcon Surgical, Fort Worth, TX) was inserted with the injector system through the 2.8 mm incision into the bag. The limbal wound was not sutured [Figure 1].

**Table 1:** Surgical parameters

	Power	Mode	US tip	Aspiration rate (cc)	Vacuum
Torsional	70%-100% Torsional amplitude	OZil continuous mode, linear pedal control	Kelman ABS 0.9 mm 30° bevel	43	300
Longitudinal	40-60%	Pulse mode (10 pulses/second)	Straight, ABS, 0.9 mm	38	400

No tests applied; only descriptive

The main outcome parameters were mean UST and mean cumulative dissipated energy (CDE). The UST represents the number of seconds the foot pedal remained in the third position. The mean CDE power indicates the mean percentage of power spent during the UST. (CDE = mean US power × UST) The UST and CDE values in torsional and phaco modes were automatically calculated by the device and displayed on the monitor of the phaco machine. In torsional mode, the CDE was calculated as follows: Torsional amplitude × torsional time × 0.4. The frequency of the phaco tip in torsional mode was 80% of that in standard phacoemulsification (32 Khz in torsional versus 40 Khz in standard phacoemulsification), and the stroke distance of the phaco tip in torsional mode was half that in standard phacoemulsification. This justified the coefficient of 0.4.

The patients were seen one day, seven days and 30 days postoperatively. The postoperative best corrected visual acuity (BCVA), degree of corneal edema and intra and post-operative complications were all documented. Corneal edema was graded as trace; where there was minimal corneal clouding and thickening in relation to incision sites, mild - corneal clouding and thickening affecting less than 25% of the cornea with no Descemet's folds and clear iris details, moderate-corneal clouding and thickening affecting more than 25% of the cornea with few Descemet's folds and hazy iris details and severe-corneal clouding and thickening affecting more than 50% of the cornea with more Descemet's folds and no view of iris details. Central corneal thickness was measured with Visante OCT 1000 (Carl Zeiss Meditec) anterior segment imaging.

The SPSS 13.0 (SPSS Inc. Chicago, Illinois) was used. The independent-samples *t* test, was used to compare decimal visual acuity, UST, CDE and CCT. Chi-square test was used to compare qualitative variables while the unpaired *t*-test was used to compare quantitative variables between groups. Test was considered significant (S) if *P* < 0.05, highly significant (HS) if *P* < 0.01 and non significant (NS) if *P* > 0.05.^7^

## RESULTS

A total of 200 eyes were enrolled in this study (156 patients); 100 in the conventional US group and 100 in the torsional US group according to LOCS II classification. The mean age was 67.9 years ± 3.9.

**Figure 2:**
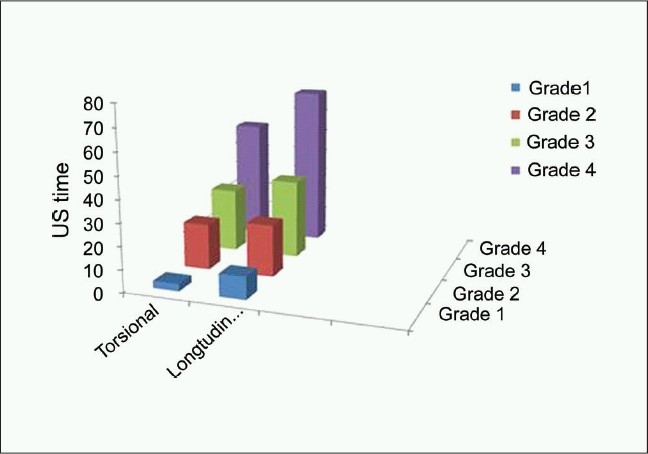
The US time in the torsional and longitudinal groups in various lens nucleus density grades

**Figure 3:**
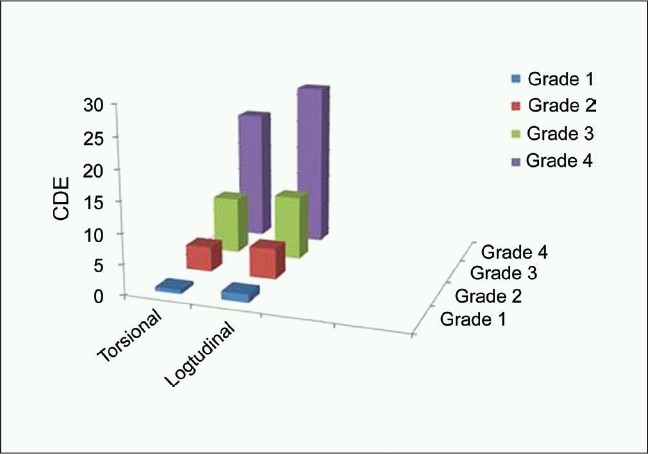
Cumulative dissipated energy in the 2 groups in different nucleus density grades

The differences in UST and CDE between subgroups of nucleus hardness were statistically significant (*P* < 0.01). The UST and CDE consistently increased in eyes with higher grades of nucleus density [Tables 2 and 3]. The mean UST and CDE were lower in the torsional group than in the US group for all grades of nucleus densities. The difference was significant for Grade-1 and Grade-4. Tables 2,3 and Figures 2 and 3 illustrate the different in the UST and CDE between the two groups at different nucleus densities.

**Table 2:** Ultrasound time (seconds) in two groups

	Nucleus density grade			
	
	Grade-1 N = 87	Grade-2 N = 38	Grade-3 N = 38	Grade-4 N = 37
Torsional	3.25 ± 0.4 N = 45	20.43 ± 5.3 N = 17	28.41 ± 12.7 N = 20	53.19 ± 27.3 N = 18
Longitudinal	10.12 ± 3.8 N = 42	23.22 ± 11.3 N = 21	35.14 ± 15.5 N = 18	71.24 ± 11.8 N = 19
*P*	t = 11 *P < *0.01 (HS)	t = 1.3 *P > *0.05 (NS)	t = 1.9 *P > *0.05 (NS)	t = 9.5 *P < *0.01 (HS)

**Table 3:** Cumulative dissipated energy in two groups

	Nucleus Density Grade			
	
	Grade-1	Grade-2	Grade-3	Grade-4
Torsional	0.65 ± 0.2	4.16 ± 2.2	9.33 ± 11.8	22.02 ± 9.7
Longitudinal	1.35 ± 0.1	5.12 ± 1.1	10.61 ± 3.2	27.571 ± 6.6
*P*	t = 2.9	t = 1.8	t = 1.6	t = 2.6
	*P* < 0.01	*P* > 0.05	*P* > 0.05	*P* < 0.05

On day one, the mean BCVA for all grades was 0.61 ± 0.13 decimals in the US group and 0.67 ± 0.11 decimals in the torsional group. This difference was statistically significant (*P* < 0.05).

By 30 days, the mean BCVA for all grades was 0.94 ± 0.22 decimals in the US group and 1.0 ± 0.12 decimals in the torsional group the difference was not statistically significant. (*P* > 0.05).

The frequency of severe central corneal edema and Descemet's striae was higher in the US group than in the torsional group [Table 4]. On day one, the difference between the two groups was statistically significant (*P* < 0.05). By 30 days, there was no corneal edema in either group. Except for corneal edema, no intraoperative or postoperative complications were noted in either group.

**Table 4:** Comparison of post operative corneal edema at day one and one month in both groups

Parameters	Corneal Edema Grading				
	
	Non	Trace	Mild	Moderate	Severe
	(0)	(1)	(2)	(3)	(4)
Longitudinal group (Day-1)	65	19	5	10	1
Torsional group (Day-1)	81	12	2	5	0
Longitudinal group (month-1)	100	0	0	0	0
Torsional group (month-1)	100	0	0	0	0

*P* value at day 1; X^2^ = 12.6; *P* < 0.05

## DISCUSSION

In recent years, technological developments have led to advances in cataract surgery and new methods to reduce US energy have been achieved. Examples include non ultrasonic energy such as sonic frequencies, NeoSoniX-generated tip rotation,^8^–^10^ and pulse water-jet technology. Restricting energy to fractions of second pulses or bursts,^11^,^12^ and millisecond-level microburst,^13^ tip design,^14^ and specific chopping technique^15^–^20^ are favorable to lower energy. For the lens to be removed efficiently and safely, the risk of ultrasound induced endothelial cell loss should be minimized. Reducing phacoemulsification energy and time are the main objectives of future improvement.

A recent study by Davison compared surgeon-generated tip travel and surgical time of longitudinal and torsional phacoemulsification with straight and angled tips. He found that the angled tip/ torsional US combination reduced tip travel by more than 40% compared with the straight tip/longitudinal US. He stated that shorter cumulative tip travel and less procedure time imply increased nuclear followability, fewer reacquisition movements, and increased phacoemulsification efficiency and safety.^21^

In this study we compared the safety and efficacy of torsional mode with longitudinal phacoemulsification in different grades of nucleus densities. Our results demonstrate that torsional phacoemulsification produces a safe and efficient mode of phacoemulsification than longitudinal mode with reduced mean UST and CDE in all grades of nucleus densities. Our results are supported by results obtained by Yizhi Liu *et al*.^2^ Energy saving (as determined by CDE) was significant in nucleus Grade-1 and Grade-4 following torsional phacoemulsification. This was reflected in the absence or traces of corneal edema in most patients on the first day and only a few patients with mild or moderate corneal edema that resolved within one week of adequate treatment following torsional phacoemulsification.

As mentioned before, torsional phacoemulsification causes less repulsion of nuclear materials than conventional phacoemulsification, so lens materials stays at the tip most of the time. As a consequence, in our study less vacuum level was used with torsional mode compared to the longitudinal mode, this counteracts surge and the outcome is a safer surgery.

The mean BCVA at day one was significantly better in the torsional group which was attributable to less corneal edema secondary to less UST and CDE. However, at 30 days postoperative, the mean BCVA was nearly the same in both groups with no statistically significant differences. This emphasize that torsional phacoemulsification has better visual outcomes in the early postoperative period.

One of the limitations of this study is that we did not evaluate the effect of surgery on the corneal endothelial cell count. The corneal thickness measurement by anterior segment OCT was used as a surrogate for endothelial cell function thus indirectly evaluating the effect of surgery on corneal endothelium. Further, other studies have demonstrated clinically significant decrease in endothelial cell loss with torsional phacoemulsification mode in comparison to conventional phacoemulsification.^2^The loss of endothelial cell count also correlated with the size of incision, where 2.2 mm incision with phacomeulsification sleeve (Ultrasleeve^TM^) produced clinically significant less endothelial cell loss than standard 2.8 mm incision with microsleeve.^22^

## CONCLUSION

Torsional mode phacoemulsification is an effective and safe technique for cataract surgery. It has the advantage of reducing UST, as well as effective energy used with earlier visual rehabilitation that makes torsional mode phacoemulsification the technique of the future.
